# Ascarosides coordinate the dispersal of a plant-parasitic nematode with the metamorphosis of its vector beetle

**DOI:** 10.1038/ncomms12341

**Published:** 2016-08-01

**Authors:** Lilin Zhao, Xinxing Zhang, Yanan Wei, Jiao Zhou, Wei Zhang, Peijun Qin, Satya Chinta, Xiangbo Kong, Yunpeng Liu, Haiying Yu, Songnian Hu, Zhen Zou, Rebecca A. Butcher, Jianghua Sun

**Affiliations:** 1 State Key Laboratory of Integrated Management of Pest Insects and Rodents, Institute of Zoology, Chinese Academy of Sciences, Beijing 100101, China; 2 Department of Chemistry, University of Florida, Gainesville, Florida 32611, USA; 3 Department of Forestry, Northeast Forestry University, Haerbin 150040, China; 4 The Research Institute of Forest Ecology, Environment and Protection, The Chinese Academy of Forestry, Beijing 100091, China; 5 Forestry Academy of Jiangsu Province, Nanjing 211153, China; 6 CAS Key Laboratory of Genome Sciences and Information, Beijing Institute of Genomics, Chinese Academy of Sciences, Beijing 100101, China

## Abstract

Insect vectors are required for the transmission of many species of parasitic nematodes, but the mechanisms by which the vectors and nematodes coordinate their life cycles are poorly understood. Here, we report that ascarosides, an evolutionarily conserved family of nematode pheromones, are produced not only by a plant-parasitic nematode, but also by its vector beetle. The pinewood nematode and its vector beetle cause pine wilt disease, which threatens forest ecosystems world-wide. Ascarosides secreted by the dispersal third-stage nematode L_III_ larvae promote beetle pupation by inducing ecdysone production in the beetle and up-regulating ecdysone-dependent gene expression. Once the beetle develops into the adult stage, it secretes ascarosides that attract the dispersal fourth-stage nematode L_IV_ larvae, potentially facilitating their movement into the beetle trachea for transport to the next pine tree. These results demonstrate that ascarosides play a key role in the survival and spread of pine wilt disease.

## Abstract (web summary)

Many species of nematodes use pheromones called ascarosides to coordinate their behaviour and development. Here, Zhao *et al*. demonstrate that the beetle vector of the pinewood nematode (*Bursaphelenchus xylophilus*) also uses and responds to ascarosides in its interactions with the nematodes.

## Graphical abstract

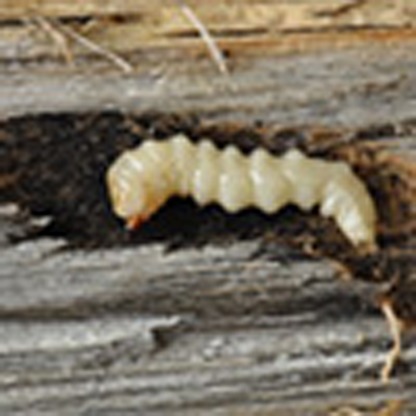

Pine wilt disease (PWD), which is caused by the pinewood nematode (PWN), *Bursaphelenchus xylophilus*, has devastated millions of hectares of pine forests in parts of Asia and Europe and has been placed under global quarantine^1^,^2^. PWN is transported to host pine trees by its insect vector, the *Monochamus* beetle, during the summer^3^. Inside the pine tree, the nematode develops rapidly through four larval stages (L_1_–L_4_) to the reproductive adult and weakens the pine tree host. *Monochamus* beetles lay eggs on the weakened pine tree in autumn, and the resulting beetle larvae develop within the pine tree. However, under unfavourable conditions such as low food and temperature in the winter, the nematode enters the dispersal phase of its life cycle, by molting from L_2_ into dispersal juveniles (L_III_)^4^. During the following spring, L_III_ are attracted to the beetle larvae and aggregate around their pupal chambers^2^,^5^. Once the beetle larvae become late pupae or early adults, the L_III_ larvae are induced to develop into fourth-stage dispersal juveniles (L_IV_). These L_IV_ then enter the tracheal system of the beetle for transport to a new pine tree host^2^,^5^.

Chemical signals play a key role in mediating interspecific interactions between PWN, its vector beetle and the pine tree host. The aggregation of L_III_ larvae around the beetle pupal chambers is triggered by terpene signals produced by the beetles^6^ as the beetles prepare to develop into adults. The L_III_–L_IV_ molt in PWN is stimulated by fatty acid ethyl esters produced by beetles, specifically during the late pupa and early adult stages of the beetle, ensuring that the nematodes reach the dispersal L_IV_ stage as the newly eclosed adult beetle prepares to leave the tree^7^.

Chemical signals also play an important role in mediating intraspecific interactions within populations of nematodes. Many species of free-living and parasitic nematodes coordinate their behaviour and development by secreting a family of pheromones called the ascarosides^8^,^9^,^10^,^11^,^12^. The ascarosides are derivatives of the 3, 6-dideoxy-L-sugar ascarylose modified with fatty acid-derived side chains, and they can be designated according to the number of carbons in their side chains^12^,^13^,^14^,^15^. The ascarosides have been shown to affect dauer formation, sexual attraction, aggregation, dispersal and olfactory plasticity in nematodes^8^,^10^,^11^,^13^,^14^.

In this study, we show that ascarosides are produced not only by PWN, but also, surprisingly, by its vector beetle. We demonstrate that these ascarosides mediate interspecific interactions between PWN and the beetle *M. alternatus*. Ascarosides produced by the beetle at low temperatures enable the beetle to postpone larval development until environmental temperatures increase. Ascarosides produced by the L_III_ larvae as they aggregate around the beetle's pupal chamber help to promote the metamorphosis of the beetle into the adult stage. Ascarosides produced by the beetle are attractive to the L_IV_ larvae and might direct the movement of the larvae into the tracheal system of the beetle. Knowledge of these interspecific signals could potentially shed light on the driving forces behind the spread of PWN and inspire better approaches to preventing transmission.

## Results

### Ascarosides produced by PWN promote pupation of the beetle

**Figure 1:**
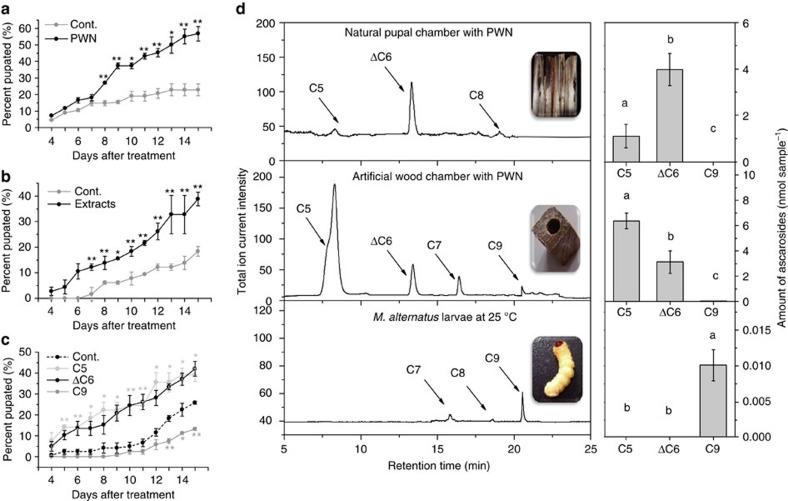
Effects of PWN L_III_ larvae and ascarosides on beetle development. (**a**) The effect of PWN L_III_ larvae on the time to metamorphosis of the beetles (*n*=20). Values are expressed as mean±s.e.m. of three independent experiments. *P<0.05 versus control and **P<0.01 versus control (Student's *t*-test, unpaired, two-tailed). (**b**) The effect of ethanol extracts from chambers with numerous PWN L_III_ larvae on the time to metamorphosis of the beetle (*n*=20). Values are expressed as mean±s.e.m. of three independent experiments. *P<0.05 versus control and **P<0.01 versus control (Student's *t*-test, unpaired, two-tailed). (**c**) Effects of ascarosides on the time to metamorphosis of the beetle. Asc-ΔC6 was tested at 3.97 nM (the concentration in each natural chamber with about 5,000 L_III_ larvae), asc-C5 at 6.37 nM (the concentration in each artificial chamber with about 5,000 L_III_ larvae), and asc-C9 was tested at 0.05 nM (the concentration in each beetle larva at 4 °C; *n*=20). Values are expressed as mean±s.e.m. of three independent experiments. *P<0.05 versus control and **P<0.01 versus control (Kruskal–Wallis nonparametric test, Mann–Whitney *U* test). (**d**) Ascarosides detected using LC–MS/MS in extracts from natural chambers with PWN, artificial chambers with PWN and the beetle surface (*n*=20). Values are expressed as mean±s.e.m. of five independent experiments. *P*<0.05 (ANOVA, Tukey's multiple comparison test). Labels with different letters are significantly different at *P*=0.05.

In field observations over three seasons, we found that beetle larvae associated with PWN tended to pupate and become adults earlier than those without the nematodes (Supplementary Table 1). Therefore, we decided to investigate the effect of the population density of PWN L_III_ larvae on the development of the beetle larvae under controlled laboratory conditions. The presence of high numbers of L_III_ larvae in artificial chambers dramatically decreased the time required for the beetle pupation (Fig. 1a). The percentage of beetle larvae that had pupated was ∼30% higher in the presence of the L_III_ larvae than in control samples at 15 days (Fig. 1a). Conversely, the time required for the beetle to develop from the early pupa to the adult stage was not significantly affected by the presence of the L_III_ larvae (Supplementary Fig. 1a). Overall, the presence of L_III_ larvae decreased the time required for the beetles to develop from larvae to adults by about 10 days (Supplementary Fig. 1b), similar to what was observed in the field (Supplementary Table 1). Importantly, ethanol extracts generated from natural chambers containing PWN L_III_ larvae promoted beetle pupation (Fig. 1b). This result suggests that chemical signals produced by PWN L_III_ larvae promote beetle pupation.

To investigate this hypothesis, we analysed for the presence of ascarosides in ethanol extracts from different samples including: natural chambers containing L_III_ larvae, artificial chambers inoculated with L_III_ larvae and the fungus *Botrytis cinerea* (on which PWN feed), artificial chambers with only *B. cinerea* or the PWN-associated fungus *Sporothrix* sp.1 (ref. 2), and the surface of beetle larvae that had been raised on an artificial diet in a sterile environment. The presence of asc-C5 (ascr#9) and asc-ΔC6 was detected in the natural and artificial chambers containing PWN L_III_ larvae, but not on the surface of the beetle larvae (Fig. 1d). Surprisingly, we detected asc-C9 (ascr#10) on the surface of beetle larvae (Fig. 1d). No ascarosides were detected in the artificial chambers inoculated with only *B. cinerea* or *S.* sp. 1 (Supplementary Fig. 2).

To determine whether these ascarosides affected the development of the vector beetle, we treated fifth instar beetle larvae with each of the compounds at the same concentrations as produced in a chamber with PWN or by a single beetle. At these concentrations, asc-ΔC6 and asc-C5 promoted beetle pupation, while asc-C9 did not (Fig. 1c). Therefore, the ascarosides secreted by PWN promote beetle pupation.

**Figure 2:**
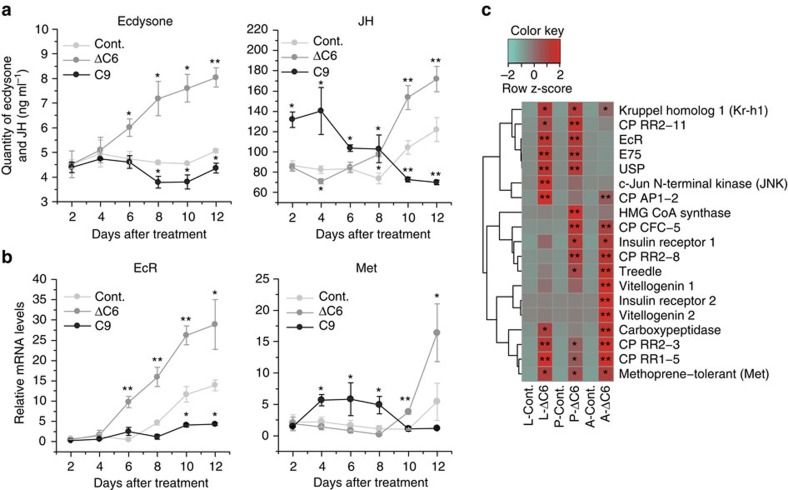
Effects of ascarosides on hormone production and gene expression. (**a**) Effects of asc-ΔC6 and asc-C9 on ecdysone and JH production in fifth instar beetle larvae. Values are expressed as mean±s.e.m. of three independent experiments. *P<0.05 versus control and **P<0.01 versus control (Kruskal–Wallis nonparametric test, Mann–Whitney *U* test). (**b**) Effects of asc-ΔC6 and asc-C9 on gene expression of *EcR* and *Met* in fifth instar beetle larvae by qPCR. Values are expressed as mean±s.e.m. of three independent experiments. *P<0.05 versus control and **P<0.01 versus control (Student's *t*-test, unpaired, two-tailed). (**c**) Hierarchical clustering of gene expression of gene cohorts related to autogeny, JH and ecdysone, and cuticle proteins in larvae, pupae and adults by qPCR (*n*=3). L-Cont.: control larvae after 12 days; L-ΔC6: larvae after 12 days treatment with asc-ΔC6; P-Cont.: control pupae after 1 day; P-ΔC6: pupae after 1 day treatment with asc-ΔC6; A-Cont.: control adults after 1 day; A-ΔC6: adults after 1 day treatment by asc-ΔC6. Values are expressed as mean±s.e.m. of three independent experiments. *P<0.05 versus control and **P<0.01 versus control (Student's *t*-test, unpaired and two-tailed).

In insects, two major developmental hormones, ecdysone and juvenile hormone (JH), are involved in the metamorphosis^16^,^17^. Ecdysone binds to a heterodimer of ecdysone receptor (EcR) and ultraspiracle (Usp)^16^,^17^. Downstream signalling induces growth arrest and pupation^17^. JH, on the other hand, maintains the status quo until the larvae obtain sufficient nutrients to trigger metamorphic transition^18^. Before pupation, ecdysone and related genes are up-regulated, and then the JH pathway is also up-regulated^19^,^20^. To investigate how ascarosides regulate beetle growth and maturation, we measured the concentration of ecdysone and JH, as well as the expression levels of several genes involved in JH and ecdysone signalling pathways, in beetles treated with ascarosides (Fig. 2a,b and Supplementary Fig. 3a). The treatment of fifth instar larvae with asc-ΔC6 led to an increase in the concentration of ecdysone starting around 6 days after treatment (Fig. 2a). Accordingly, asc-ΔC6 treatment also up-regulated (from 6 to 8 days after treatment) the expression of *EcR* and *USP*, as well as *E75* (ref. 21), an early ecdysone-inducible gene (Fig. 2b and Supplementary Fig. 3a). In addition, cuticle proteins in larvae and late-stage pupae treated with ascarosides were also up-regulated, possibly promoting the molting process^22^. These data suggest that asc-ΔC6 may promote the transition from larval to pupal development by inducing the production of ecdysone in the beetle larvae. Additionally, asc-ΔC6 treatment led to an increase in the concentration of JH and the mRNA abundance of the JH receptor, *methoprene-tolerant* (*MET*)^19^, and a key repressor of metamorphosis, *Kruppel homolog 1* (*Kr-h1*)^23^, but at a later time point, once a large fraction of larvae had pupated (Fig. 2a–c and Supplementary Fig. 3a).

To determine the effects of asc-ΔC6 on the fecundity of the beetle, we compared the number of eggs laid by control females and asc-ΔC6-treated females. The asc-ΔC6-treated females produced more eggs than control females (Supplementary Fig. 3b). Genes regulating reproduction and egg formation, including MET, insulin receptor and vitellogenin, were also significantly up-regulated in the adults treated with asc-ΔC6 compared with the control adults (Fig. 2c). Thus, asc-ΔC6 secreted by PWN promotes pupation and increases fecundity of the vector beetle.

### Ascarosides maintain the beetle in the larval stage during the winter

**Figure 3:**
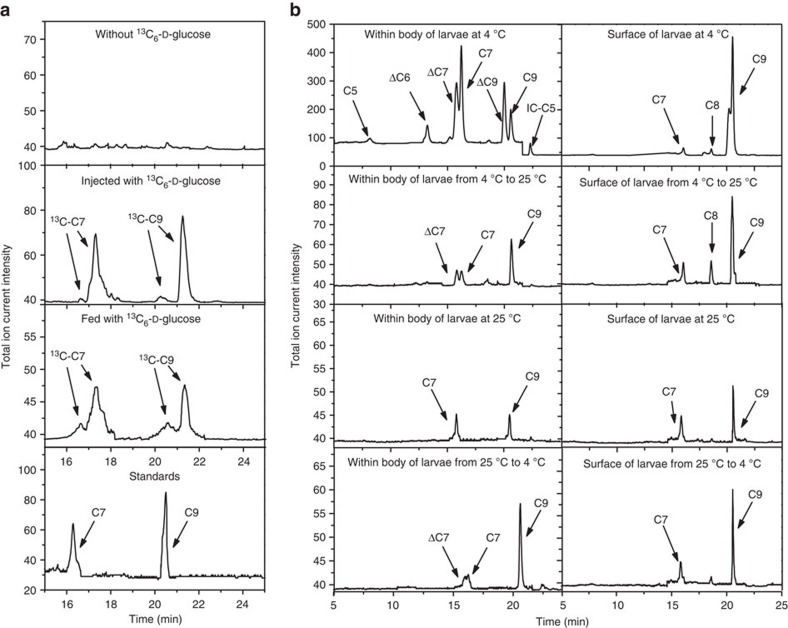
Biosynthesis of ascarosides by *M. alternatus*. (**a**) Detection of ^13^C-labelled asc-C7 and asc-C9 by LC–MS/MS in SRM mode in larvae injected or fed with ^13^C-labelled glucose (*n*=20). There were two peaks for labelled asc-C7: left arrow: asc-C7 with only sugar labelled (*m/z* 281.31), right arrow: asc-C7 with sugar labelled and side chain completely labelled (*m/z* 288.32). There were two peaks for labelled asc-C9: left arrow: asc-C9 with only sugar labelled (*m/z* 309.37), and right arrow: asc-C9 with sugar labelled and side chain completely labelled (*m/z* 318.37). The percentages of labelled asc-C7 and asc-C9 were 88 and 57%, respectively, in the injected beetles, and 39 and 22%, respectively, in the fed beetles. Deuterium labelling slightly increases the retention time of relevant ascarosides. (**b**) LC–MS/MS analysis of ascarosides inside beetles and on the surface of beetles grown at different temperatures.

To provide further evidence that *M. alternatus* beetles could produce ascarosides, we reared the beetles in a sterile environment on an artificial diet with antibiotics to eliminate any bacteria associated with the insect (Supplementary Fig. 4). We then fed them stable isotope-labelled glucose and determined whether the label was incorporated into the ascarosides. Glucose has been shown to be the precursor for ascarylose biosynthesis in bacteria (*Yersinia pseudotuberculosis*)^24^. In one labelling method, we injected ^13^C-labelled glucose into the body cavity of beetle larvae and allowed them to grow for 5 days. Alternatively, we fed the beetle larvae with an artificial diet containing ^13^C-labelled glucose for 14 days. ^13^C-labelled ascarosides, specifically asc-C7 and asc-C9, were detected, with the labelling occurring in the ascarylose sugar, as well as in the side chain (Fig. 3a).

We further examined the effect of asc-C9 on the development of the beetle. The concentration of asc-C9 on the surface of beetle larvae was 10 times higher for larvae grown at 4 °C versus 25 °C (Fig. 3b). Also, asc-C9 slows the rate of pupal formation at the same concentration as produced by a beetle larva at 4 °C (Fig. 1c). Thus, the beetle larvae could potentially produce more asc-C9 during the cold winter time to inhibit developmental progression. In spring, with higher temperatures, the production of asc-C9 may decrease, enabling developmental progression to resume. Furthermore, several ascarosides were detected inside the beetle, including asc-C7 (ascr#1), asc-ΔC7 (ascr#7), asc-ΔC9 (ascr#3) as well as asc-C9 (Fig. 3b). Similar to asc-C9, these ascarosides also suppressed the development of beetle (Supplementary Fig. 5a). Unlike asc-ΔC6 treatment, asc-C9 treatment slightly decreased the concentration of ecdysone and down-regulated the expression of *EcR*, *E75* and *USP* in fifth instar larvae (Fig. 2a; Supplementary Fig. 3a). Unlike asc-ΔC6 treatment, which induced JH biosynthesis 10 days post-treatment, asc-C9 treatment induced JH biosynthesis in fifth instar larvae at a much earlier time point (4 days post-treatment; Fig. 2a). Asc-C9 treatment also up-regulated the expression of *MET* and *Kr-h1* (Fig. 2b and Supplementary Fig. 3a). Thus, the increased production of asc-C9 at lower temperatures may delay pupation.

To investigate the effect of a temperature shift on the production of asc-C9 in fifth instar larvae, we transferred the larvae from 4 to 25 °C or from 25 to 4 °C. After 24 h, the concentration of asc-C9 on the surface and in the beetle body was reduced by a temperature upshift and was increased by a temperature downshift (Fig. 3b, Supplementary Fig. 5b), indicating that the concentration of asc-C9 changes rapidly in response to temperature shifts.

**Figure 4:**
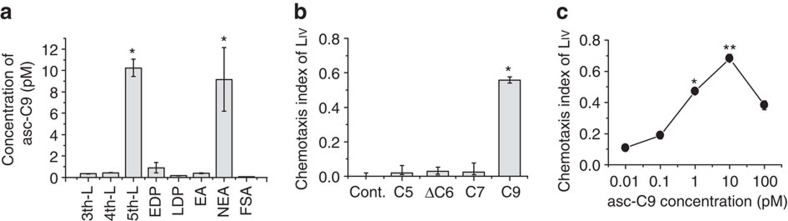
Effect of the ascarosides produced by adult beetle on PWN behaviour. (**a**) The measurement of asc-C9 on the surface of the beetle from different developmental stages, including EDP, early development pupa; EA, emerging adult; FSA, fully sclerotized adult; L, larva; LDP, late development pupa; NEA, newly eclosed adult. Values are expressed as mean±s.e.m. of five independent experiments. *P<0.05 versus the L_III_ larvae (Student's *t*-test, unpaired and two-tailed). (**b**) Effects of different ascarosides at the same concentrations as produced by a chamber or one newly eclosed adult beetle on the chemotaxis index of PWN L_IV_ larvae. Values are expressed as mean±s.e.m. of 10 independent experiments. *P<0.05 versus control (Student's *t*-test, unpaired, two-tailed). (**c**) Dosage dependent effects of asc-C9 on the chemotaxis index of L_IV_. Values are expressed as mean±s.e.m. of 10 independent experiments. *P<0.05 versus 0.01 pM and **P<0.01 versus 0.01 pM (Student's *t*-test, unpaired, two-tailed).

Ascaroside secretion by the adult beetle is attractive to the PWN L_IV_ larvae. We measured the level of asc-C9 produced by the beetle during all stages of beetle development and observed that the concentration of asc-C9 was extremely high in fifth instar larvae, decreased sharply during pupal stages, then increased sharply with the emergence of the adult, and then decreased sharply in the fully sclerotized adult (Fig. 4a). Unlike other ascarosides produced by PWN or found in natural chambers, asc-C9 could attract L_IV_ when used at the same concentration as is present on the surface of a newly eclosed adult (Fig. 4b,c). On the other hand, the concentration of asc-C9 in the natural chambers was not enough to attract the L_IV_ larvae (Supplementary Fig. 5c). L_III_ larvae were also not attracted by asc-C9 (Supplementary Fig. 5d). Therefore, asc-C9 could potentially contribute to the movement of the L_IV_ dispersal larvae into the tracheal system of the newly eclosed adult beetle for dispersal to a new pine tree host.

## Discussion

Here, we are the first to report that ascarosides are produced not only by nematodes, but also by *M. alternatus* beetles. After being reared on an artificial diet in a sterile environment, the beetles were able to incorporate stable isotope-labelled glucose into the ascarosides, demonstrating that the beetles are able to biosynthesize the ascarosides. We have shown that the production of asc-C9 by the beetle increases at lower temperatures and potentially inhibits developmental progression during the cold winter. The chemical signals which delay development or diapause in many types of insects remain unknown, but in *M. alternatus*, asc-C9 likely plays this role.

Asc-ΔC6 and asc-C5 secreted by PWN promote beetle development from the larval to the pupal stage, facilitate the spread of PWD, as well as increase the reproductive advantage of the beetle. These signals might help to synchronize the beetle's development with that of PWN. L_III_ larvae of PWN must accumulate in the tree before the beetle develops into the pupal stage, so that when the adult beetle emerges, the fatty acid ethyl esters produced by the adult can stimulate the development of the nematode from the L_III_ to L_IV_ stage^7^. These coordinated actions likely contribute to increased PWN transmission by the beetle. Our data suggest that the development and fitness of the beetle should track with the year-to-year and location-to-location differences in the density of nematodes around the natural beetle chamber. The interaction between PWN and its vector beetle could serve as a model for studying developmental synchronization during infection of a pine tree host. In the future, it will be interesting to determine whether other *Bursaphelenchus* nematode species and *Monochamus* beetle species produce ascarosides. It is possible that the native partners PWN (*B. xylophilus*) and *M. carolinensis* in North America, as well as the native partners *B. mucronatus* and *M. alternatus* in Asia, engage in ascaroside-mediated interactions. Thus, the ascaroside-mediated interactions between the non-native partners PWN and *M. alternatus* would not necessarily have had to evolve *de novo*, but could have already been in place when the two non-native partners were introduced to one another. In the future, it will also be interesting to investigate the effects of the ascarosides on *B. mucronatus*, which has been competitively displaced by PWN in Asia^2^,^25^, as well as on PWN-associated fungi and bacteria.

Taken together, our data show that the function of the ascarosides is to mediate mutually beneficial interactions between PWN and its vector beetle. Ascaroside production in PWN and its vector beetle is closely tied to environmental conditions, similar to what is seen in *C. elegans*^26^. Beetle-produced ascarosides regulated by the environmental temperature influence the beetle development, while PWN-produced ascarosides enable the accumulation of PWN in the tree to influence beetle development. Additionally, beetle-produced ascarosides synchronize the aggregation behaviour of PWN with beetle development, potentially facilitating the movement of PWN into the beetle trachea as it develops into an adult. Our findings may lead to the development of more effective approaches to controlling PWD that interfere with the development of the vector beetle or the behaviour of PWN and thus prevent further spread of the disease.

## Methods

### Field investigation

To estimate the relationship between the population density of nematodes and beetle development, the number of nematodes in the wood around the beetle pupal chamber was investigated based on a previously described^25^ method in April 2013, 2014 and 2015. At each site, thirty dead trees (which died between the previous September and the following April) with a dish (diameter at breast height, dbh) of 10–15 cm were chosen for study. One pupal chamber was randomly sampled from the southern side of the xylem in a chosen tree at breast height for each 10–20 km (ref. 25). L_III_ in ∼20 g of wood around each pupal chamber were sampled in April and July, respectively, and placed in Baermann funnels to recover nematodes, which were then counted by direct observation through a dissecting microscope^6^. The average number of nematodes around pupal chambers from each site was used to estimate the density of the nematodes. The developments of beetle larvae were investigated from thirty natural chambers chosen as mentioned above at each site in April 2013, 2014 and 2015. The percent pupation and emergence time of beetle larvae in 30 natural chambers with beetle larvae together was investigated in April of each year.

### Construction of artificial chambers

A hole (4 cm deep and 1 cm diameter) was drilled in the centre of the cut ends of *Pinus massoniana* blocks (5 cm long, 2.5 cm mean diameter). The blocks were autoclaved at 121 °C for 30 min, then inoculated with *B. cinerea* for 7 days, followed by inoculating with 4,000 propagative nematodes in aerated Erlenmeyer flasks (100 ml) and incubated at 25 °C in the dark for 2 months^6^.

### Ascaroside analysis

Ten natural chambers that contained L_III_ larvae (chosen as described above), ten artificial chambers inoculated with the fungus *Botrytis cinerea* for 7 days without PWN and 10 artificial chambers inoculated with *B. cinerea* for 7 days followed by inoculating with PWN for 30 days^6^ (most of the nematodes were in the L_III_ stage collected as described below) were cut into small pieces. The samples were placed into a 300 ml Erlenmeyer flask and enough high-performance liquid chromatography-grade ethanol to cover the wood segments was added. The flasks were shaken in a table-top shaker for 24 h at 25 °C at 150 r.p.m. (refs 26, 27, 28).

The larvae of *M. alternatus* beetles were selected from PWN-free sites, which were identified based on the absence of PWN L_III_ larvae around the beetle chambers and the absence of L_IV_ larvae being carried by adult beetles at the sites. The beetle larvae were reared in a sterile environment on an artificial diet. Nematodes were absent from the surface and gut of the beetle larvae as determined through microscopic observation. Then the whole-body surfaces of 20 live beetles at time points corresponding to previously defined developmental stages were extracted for 30 min with 20 ml of ethanol. Then the extracted 20 larvae were frozen and lyophilized to dryness (1 day) and then ground with a pestle to a fine powder. The powder was placed in a 250 ml Erlenmeyer flask using a funnel and extracted by adding 50 ml ethanol and shaking at 150 r.p.m. for 24 h (refs 26, 27, 28).

All the samples were stored at −20 °C until further needed. The samples were filtered through filter paper and concentrated to 1 ml in vacuum centrifugal concentrator. Then 150 μl of the sample was transferred to an liquid chromatography–mass spectrometry (LC–MS) vial, and the samples were stored at 4 °C until injection^26^,^27^,^28^. These experiments were repeated three times, and the ascarosides were analysed by LC–MS/MS. Synthetic ascarosides were synthesized according to previously published methods^29^,^30^. In brief, 2, 4-di-O-benzoylascarylose was synthesized according to the method reported by Jeong *et al*.^29^. To synthesize asc-C5, 2, 4-di-O-benzoylascarylose was coupled to (*R*)-5-hexen-2-ol using BF_3_-Et_2_O, followed by oxidation of the products with KMnO_4_ (ref. 29) and deprotection with LiOH (ref. 30). To synthesize asc-ΔC6, 2, 4-di-O-benzoylascarylose was coupled to (*R*)-4-penten-2-ol using BF_3_-Et_2_O, and the product was then subjected to cross metathesis with methyl acrylate using Grubbs 2^nd^ generation ruthenium catalyst, followed by deprotection with LiOH (ref. 30). To synthesize asc-C9, 2, 4-di-O-benzoylascarylose was coupled to (*R*)-7-octen-2-ol using BF_3_-Et_2_O, and the product was then subjected to cross metathesis with methyl acrylate using Grubbs 2nd generation ruthenium catalyst, followed by hydrogenation using Pd/C and then deprotection with LiOH (ref. 30).

### Mass spectrometry

The chemical analysis was performed using an Agilent 1,290 Infinity LC/6460 triple quadrupole MS, equipped with a ZORBAX SB-Aq column (2.1 × 100 mm, 3.5 μm, Agilent, USA) and operated in the selected-reaction monitoring (SRM) mode, in which a specific precursor ion is selected, fragmented and specific product ion is monitored (*m/z* 73 for the ascarosides in the negative mode). Five or 10 μl of the samples were injected into the LC–MS/MS using a column oven temperature of 60 °C and a flow rate of 0.25 ml min^−1^ with a gradient of solvent A (0.1% formic acid+2% methanol) and solvent B (methanol), starting at 8% B for 3 min, followed by a linear gradient to 100% B over 28 min, then returning to 8% B over 5 min. Separate analyses were performed to quantify the content of asc-C5, asc-ΔC6, asc-ΔC7, asc-C7, asc-ΔC9 and asc-C9 in the negative ion mode^26^,^27^,^28^(the characteristic product ion monitored is *m/z* 73; asc-C5 *m/z*: 247.2; asc-ΔC6 *m/z*: 259.2; asc-ΔC7 *m/z*: 273.2; asc-C7 *m/z*: 275.32; asc-ΔC9 *m/z*: 301.2;asc-C9 *m/z*: 303.2). The concentration of the ascarosides in each beetle or chamber was then determined using calibration curves. Standard curves were prepared for all ascarosides using synthetic compounds before analysis.

### *M. alternatus* development assays in the presence of PWN

To test the effect of PWN on the development of vector beetle, fifth instar larvae with weights ranging from 300 to 500 mg were selected from PWN-free sites as a population in April 2014, and reared in the laboratory on an artificial diet, and the absence of nematodes contamination was confirmed. The larvae were divided randomly into different experimental groups. Artificial chambers including ∼5,000 L_III_ nematodes were constructed using pine blocks^6^. Each chamber held one larva. Twenty larvae were used in each replicate. The developmental state of beetles was observed once every 24 h until the larvae pupated^18^.

To test the effects of ethanol extracts of artificial chambers and ascarosides on the development of *M. alternatus*, artificial diets were used. 200 g of sawdust and 15 g of agar were added to 550 ml of distilled water and mixed. 400 μl of sterilized water (control), dried ethanol extract in water or ascarosides were added. Twenty larvae were used in each replicate. The developmental state of the beetle was observed once every 24 h until the larvae pupated. The experiment was repeated three times independently. Asc-ΔC6 was tested at 3.97 nM (the concentration in each natural chamber with about 5,000 L_III_), asc-C5 at 6.37 nM (the concentration in each artificial chamber with about 5,000 L_III_), and asc-C9 at 0.1 nM (the concentration in each beetle larva at 4 °C).

### Ecdysone and JH measurement

Haemolymph was collected by puncturing the abdomen of the beetles untreated or treated with asc-ΔC6 and asc-C9 and diluting 100-fold with phosphate buffer saline. Ecdysteroids were detected with the human ecdysone ELISA kit (Nuoyajie Corporation, Beijing, China) according to the instructions. The experiment was repeated three times. LC–MS/MS tandem mass spectrometry was used to quantify the JH level^31^. In brief, beetle samples were weighed and used to collect haemolymph by puncturing the abdomen. Each micro-glass tube stored 50–100 μl of haemolymph with three replicates for different developmental times. *S*-(+)-methoprene (Acros Organics) and JH III (Sigma-Aldrich, purity ⩾65%) were dissolved in methanol. A total of 100 ng of an internal standard (*S*-methoprene) was added to each of the beetle haemolymph samples. Additional methanol was added to make the ratio of haemolymph: methanol, 1:10. The mixture was vortexed briefly and allowed to stand at room temperature for at least 20 min. After centrifuging, the upper methanol layer was removed and loaded onto a ProElut Al-N column and collected in a new 2 ml vial. The extract was concentrated to 100 μl using a stream of nitrogen gas, transferred to a new glass insert and further concentrated to 5–10 μl. Reversed phase LC–MS/MS was carried out using a Q-TOF LC–MS (Agilent Technologies). A reversed phase column (2.1 × 50 mm, 1.8 μm, ZORBAX, Eclipse XDB-C_18_) was used at a flow rate of 2 ml min^−1^ using water with 1% formic acid (solvent A) and acetonitrile (solvent B). The column was equilibrated with 10% B, and the gradient was started at 10% B, increased to 60% B over 5 min, followed by an increase to 80% B over 3 min, held at 80% B for 2 min, increased to 90% B over 3 min, followed by an increase to 100% B over 9 min and followed by a decrease to 10% B over 5 min. A total separation time of 25–35 min was used. Injection volume was 1–2 μl.

### ^13^C-labelled glucose treatment of beetles

Fifth instar larvae (∼300 mg) were selected from PWN-free sites and reared in a sterile environment on an artificial diet with antibiotics including streptomycin sulfate, ampicillin sodium salt, tetracyline HCl and nystatin at a concentration of 5 mg antibiotic per g diet for 96 h (ref. 32). To further confirm the beetles used in this experiment are free of any nematodes, we sampled and checked for the presence of nematodes on the beetle surface or in the beetle gut using microscopic observation. Associated bacteria were also absent (Supplementary Fig. 4), as determined by counting colony forming units (cultured from the gut and epidermis of the beetles on tryptic soy agar for 12 h)^33^. A 100 μg μl^−1^ solution of ^13^C_6_-D-glucose (Sigma, USA) in insect physiological saline^34^ was prepared. For the injection experiment, 5 μl of ^13^C_6_-D-glucose was injected into the body cavity of each larva with a Hamilton model 7,000 modified microliter syringe. Sixty larvae were injected and then fed for 5 days at 4 °C. Twenty larvae were collected to analyse the labelled ascarosides on the larvae surfaces. For the feeding experiment, 60 larvae were maintained for 2 weeks on an artificial diet containing ^13^C_6_-D-glucose (10 mg/2 g diet/larva). Then 20 larvae were frozen, lyophilized and then ground with a pestle to a fine powder. The powder was placed in a 250 ml Erlenmeyer flask using a funnel and extracted by adding 50 ml ethanol and shaking at 150 r.p.m. for 24 h. The chemical analysis was performed using an Agilent 1,290 Infinity LC/6460 triple quadrupole MS, equipped with a ZORBAX SB-Aq column (2.1 × 100 mm, 3.5 μm, Agilent, USA) and operated in SRM mode, as described above. Separate analyses were performed for the content of the labelled asc-C7 and asc-C9 in the negative ion mode (the characteristic product ion is *m/z* 76; labelled asc-C7 *m/z*: 281.2 for sugar labelled, 285.2 for sugar labelled and side chain partially labelled and 288.2 for sugar labelled and side chain completely labelled; labelled asc-C9 *m/z*: 309.2 for sugar labelled, 313.2 for sugar labelled and side chain partially labelled, and 318.2 for sugar labelled and side chain completely labelled).

### Gene expression analysis

According to methods used in the *M. alternatus* development assays, the beetle larvae were selected from PWN-free sites as a population in April, 2014, and reared on an artificial diet, and the absence of nematode contamination was confirmed. RNA was extracted from the whole body of each beetle treated with or without ascarosides at different developmental times with three replicates for each experiment. The samples were checked by 16S rDNA sequence analysis, and only microbial-free beetles were chosen for further quantitative PCR (qPCR) analysis. Total RNA was extracted from dissected adult beetles using TRIzol Reagent (Invitrogen, USA) according to the manufacturer's instructions. The RNA concentration and integrity was assessed using a NanoDrop ND-1000 spectrophotometer (NanoDrop Technologies, Inc., USA). The A_260_/A_280_ ratio of the RNA was between 1.8 and 2.0. mRNA was purified using Dynabeads mRNA Purification Kit (Ambion, Life technology). About 1 μg of mRNA was used as template to produce complementary DNA (cDNA) using the superscript II reverse transcriptase (Invitrogen, USA). The genes of interest were each cloned by PCR from cDNA libraries from the beetle^35^. The available putative developmental, reproductive and cuticle protein-related genes from *Tribolium castaneum* were used as template to search the *M. alternatus* transcriptome unigene sequence collection using tBLASTX at a cutoff E-value of 0.1. The deduced amino acid sequences were analysed by Pfam, Prosite, and SMART to detect conserved domain structures required for specific enzymatic function. The genes of interest were each cloned by PCR from cDNA libraries from the beetle. The cDNA libraries, representing mRNA samples of adult beetles were constructed and sequenced by high throughput Illumina techniques. The clean reads were *de novo* assembled to produce contigs using Trinity software (version 2013) with the default parameters^35^. CD-hit was used to reduce redundancy with the following parameters: sequence identity threshold (-c 0.95) and length difference cutoff (-s 0.9)^36^. After removing bacterial, fungal and nematode sequence, a total of 55,059 contigs were obtained to make unigene 1.0 (N50: 2516, bp). The longest contig contained 28,458 bases. All assembled *M. alternatus* unigenes were annotated based on the BlastX results against the non-redundant protein database (Nr) at NCBI ( http://www.ncbi.nlm.nih.gov/) and Swiss-prot database with an E-value<0.1. qPCR was performed in triplicate for each sample using SYBR PrimeScript RT-PCR Kit (TaKaRa, China) on an MX3000P Thermal cycler (Stratagene, USA)^37^. The primers used in this study are included in Supplementary Table 2. Thermal cycling of qPCR was performed at 95 °C for 30 s, followed by 40 cycles of 95 °C for 5 s, 60 °C for 30 s and 72 °C for 30 s. *β-actin* and *α-tublin* of *M. alternatus* were used as internal controls and *β-actin* was selected as the most stable one for normalization. The expression values were calculated using the ^ΔΔ^Ct method and normalized to *β-actin* expression levels^37^.

### Quadrant chemotaxis assays

Chemotaxis to ascarosides was assessed on 10 cm four-quadrant Petri plates^38^,^39^. L_IV_ larvae were sampled from trapped beetle adults, and L_III_ larvae were sampled from natural chambers in the infested sites. Each quadrant was separated from adjacent ones by plastic spacers. Pairs of opposite quadrants were filled with 2% potato dextrose agar containing different concentrations of ascarosides or vehicle. The ascarosides in ethanol were diluted 10-fold in water. Plates containing ascarosides were made by adding the ascaroside stock solution to the plate medium before it was poured into the plates (12 ml per quadrant). These plates were dried at room temperature overnight. Control plates were treated similarly except that ethanol solutions were added to the plates, corresponding to the amount of ethanol introduced via the ascaroside stock solutions. Final ethanol concentrations of the plates were below 0.1% for all conditions. Then 200–500 L_IV_ or L_III_ larvae were washed and placed in 1 μl volume in the centre of a four-quadrant plate, and scored 30 min after treatment (a time determined based on the mobility of L_IV_ larvae in preliminary experiments). A chemotaxis index was calculated as (the number of animals on ascaroside quadrants minus the number of animals on buffer quadrants)/(total number of animals)^39^. The experiment was repeated 10 times. Asc-ΔC6 was tested at 3.97 nM (the concentration in a natural chamber with ∼5,000 L_III_), and asc-C5 at 1.02 nM (the concentration in an artificial chamber with ∼5,000 L_III_). Asc-C9 wwas tested at 5 pM (the concentration in a newly eclosed adult beetle), as well as 0.01 pM (the concentration in an artificial chamber), 0.1 and 1 pM (the concentration in a small adult beetle), 10 pM (the concentration in a large adult beetle) and 100 pM.

### Fecundity of the beetles

Numbers of eggs per female that had been either treated or not treated with asc-ΔC6 were compared. Asc-ΔC6 was tested at 3.97 nM (the concentration in each natural chamber with ∼5,000 L_III_ larvae). 20 pairs of beetles (one female and one male) were randomly placed in the 20 boxes with three fresh tree branches (10 cm long, 5 cm mean diam.) in 25 °C for more than 65 days until the females died. The number of eggs laid in the phloem of the tree branches was investigated every 5 days.

### Experimental design and statistical analysis

The sample sizes were chosen to ensure adequate power (1-*ß*=0.8) to detect the significant difference between control and treatment (*α*=0.05, two-tailed). The sample sizes of all tests provided 80–97% power to detect the differences. The numbers of insects and nematodes in the bioassays were also appropriate according to the references together with the difficulty of selecting beetle larvae from the field (one generation each year). The sample sizes for chemical analysis were ascertained according to the pre-experiments that established the amounts of ascarosides present^40^. The experiments with different treatments were investigated using the same method. A student's *t*-test or analysis of variance was used to assess the statistical significance (*P* value) of differences between experimental conditions. All data were analysed by SPSS^41^.

### Data availability

All sequence data that support the findings of this study have been deposited in GenBank with the following accession numbers: β actin (KX428475), kruppel homolog 1 (Kr-h1) (KX428476), methoprene-tolerant (Met) (KX428477), HMG CoA synthase (KX428478), EcR (KX428479), USP (KX428480), E75 (KX428481), insulin receptor 1 (KX428482), insulin receptor 2 (KX428483), c-Jun N-terminal kinase (JNK) (KX428484), vitellogenin 1 (KX428485), vitellogenin 2 (KX428486), carboxypeptidase (KX428487), CP AP1-2 (KX428488), CP RR1-5 (KX428489), CP RR2-3 (KX428490), CP RR2-8 (KX428491), CP RR2-11 (KX428492), CP CFC-5 (KX428493), tweedle (KX428494). The authors declare that all the data supporting the findings of this study are available within the article and its supplementary information files or are available from the corresponding authors on request.

## Additional information

**How to cite this article:** Zhao, L. *et al*. Ascarosides coordinate the dispersal of a plant-parasitic nematode with the metamorphosis of its vector beetle. *Nat. Commun.* 7:12341 doi: 10.1038/ncomms12341 (2016).

## Supplementary Material

Supplementary Information Supplementary Figures 1 - 5 and Supplementary Tables 1 and 2
