# Supplementary material for: Ascarosides coordinate the dispersal of a plant-parasitic nematode with the metamorphosis of its vector beetle
Source: Nat Commun. 2016 Aug 1;7:12341. doi: 10.1038/ncomms12341 (PMC4974635; doi:10.1038/ncomms12341)
Supplement: Supplementary Information — Supplementary Figures 1 - 5 and Supplementary Tables 1 and 2 [file ncomms12341-s1.pdf]

Supplementary Figures

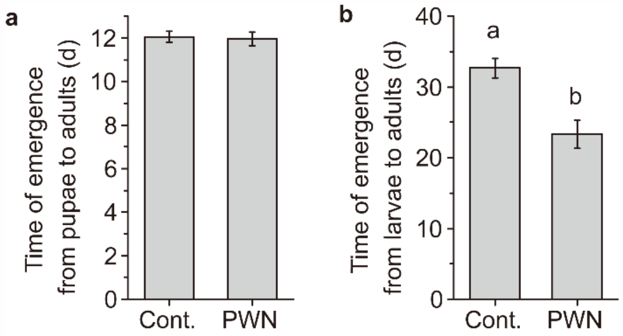

Supplementary Figure 1. The effect of numerous nematodes on the time to emergence of the beetle. (**a**) The effect of numerous dispersal L_{III} larvae on the time to emergence of the beetle from the larval stage to the adult stage (n = 20). Values are expressed as mean ± s.e.m. of three independent experiments. (**b**) The effect of numerous dispersal L_{III} larvae on the time to emergence of the beetle from the pupal stage to the adult stage (n = 20). Values are expressed as mean ± s.e.m. of three independent experiments. The significance level was set at $\alpha = 0.05$ and significant differences between groups are indicated with different letters, and statistical significance was set at a level of $P < 0.001$ (independent sample *t*-test). PWN: pinewood nematodes.

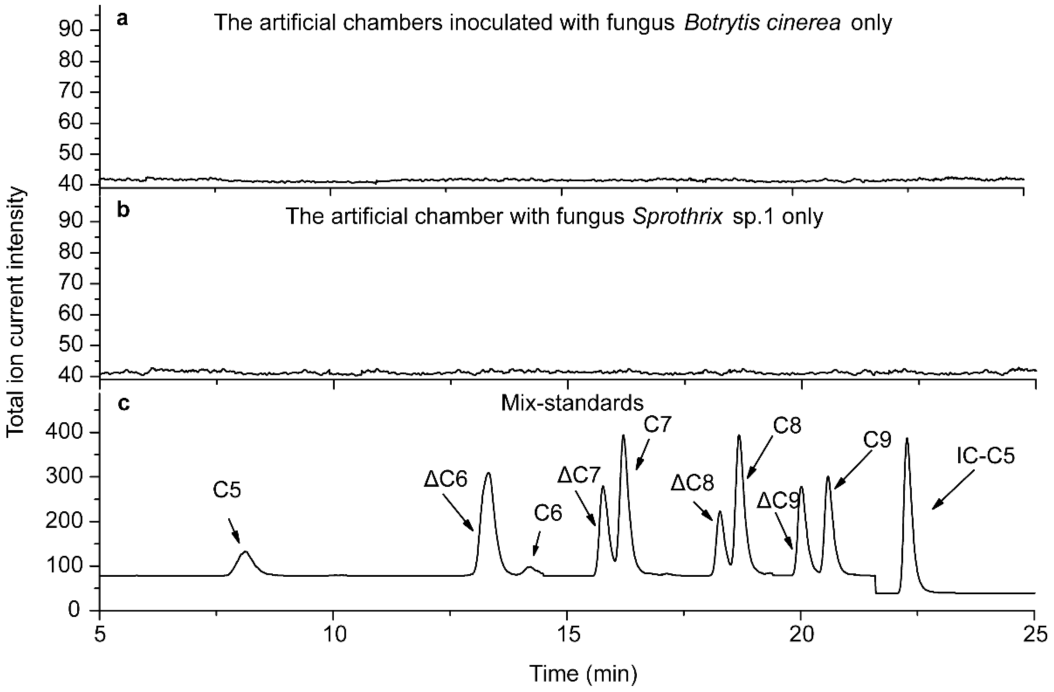

Supplementary Figure 2. Analysis of ascarosides in the artificial chambers. (a) The artificial chambers inoculated with fungus *B. cinerea* only. **(b)** The artificial chambers inoculated with *Sporothrix* sp. 1 only. **(c)** The mix-standards were as control.

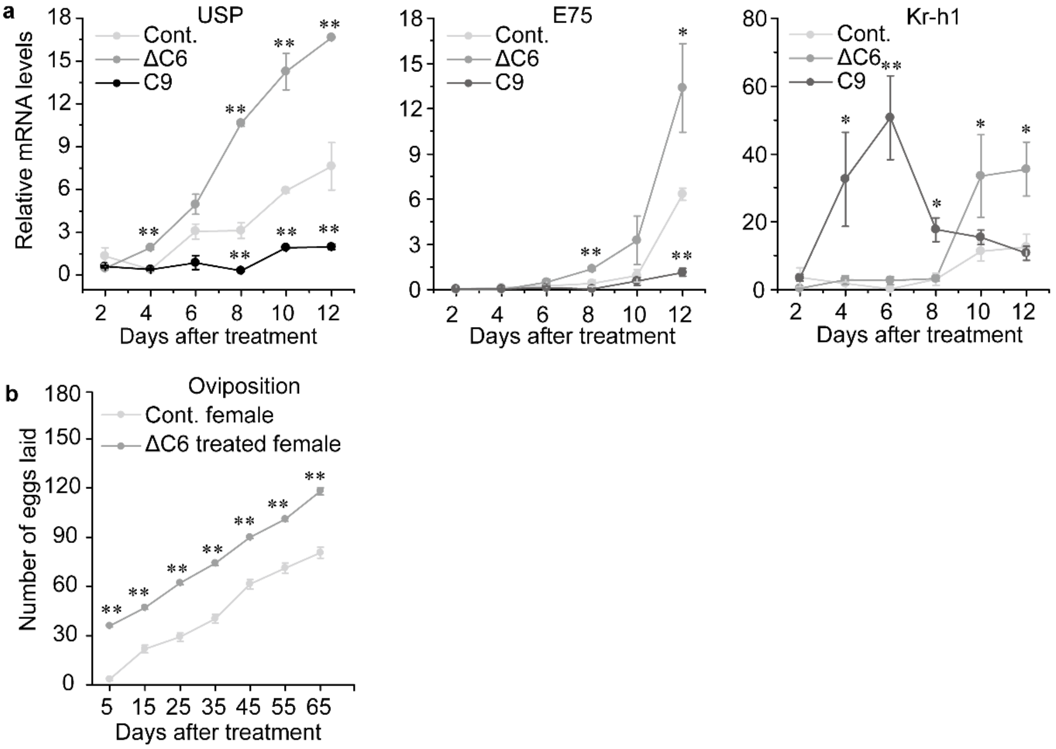

Supplementary Figure 3. Effects of asc-ΔC6 and asc-C9 on the ecdysone and JH signaling pathway and on the fecundity of the beetle. (**a**) Effects of asc-ΔC6 and asc-C9 on mRNA abundance of *USP* (left), *E75* (middle), and *Kr-h1* (right). Values are expressed as mean ± s.e.m. of three independent experiments. * $P < 0.05$ versus control and ** $P < 0.01$ versus control (student's *t*-test, unpaired, two-tailed). (**b**) Numbers of eggs per female for females that had been either treated or not treated with asc-ΔC6 (n = 20). Asc-ΔC6 was tested at 3.97 nM (the concentration in each natural chamber with about 5000 dispersal third-stage nematode larvae L_{III}). (** $P < 0.01$, multiple comparisons). Values are expressed as mean ± s.e.m. of three independent experiments. ** $P < 0.01$ versus control (student's *t*-test, unpaired, two-tailed).

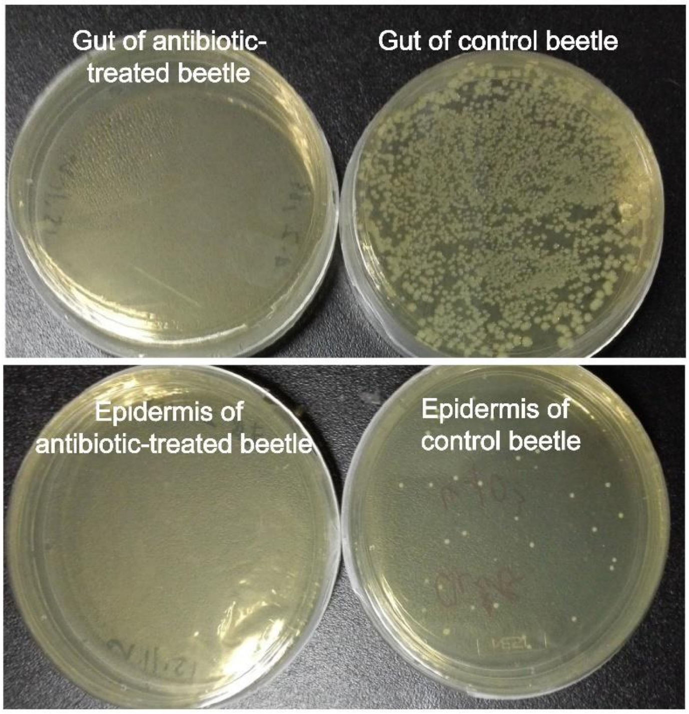

Supplementary Figure 4. Bacterial colonies from the gut and epidermis of beetles raised in the presence and absence of antibiotics.

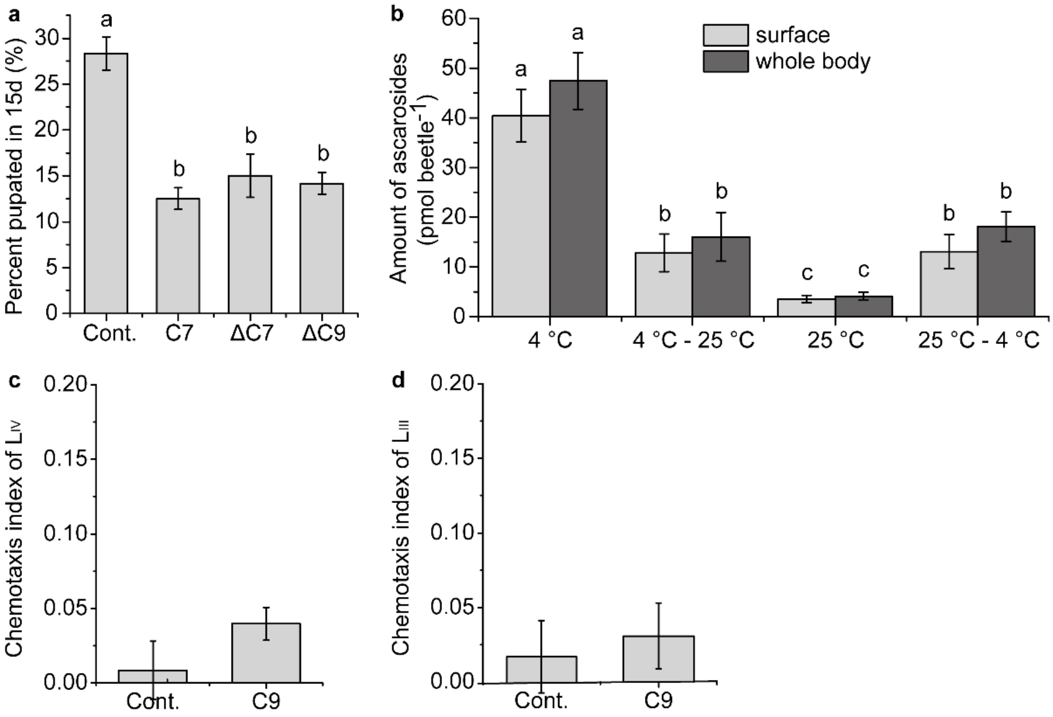

Supplementary Figure 5. Effects of ascarosides produced by the beetle internally on beetle development and the effects of beetle-produced asc-C9 on the behavior of L_{IV} and L_{III} PWN larvae. (**a**) Effects of ascarosides produced by the beetle on the time required to develop from the larval to pupal stage. Asc-C7, asc-ΔC7 and asc-ΔC9 were applied at 0.05 nM (the concentration in each beetle larva at 4 °C) (n = 20). Values are expressed as mean ± s.e.m. of three independent experiments. The data were analyzed using the one-way ANOVA test ($F_{3, 19}$ = 355.55, P < 0.001), *Post hoc* pairwise comparison were done using Bonferroni test. Labels with different letters are significantly different at P = 0.05. (**b**) The amount of asc-C9 on the surface and in the whole body of the beetle grown at different temperatures (n = 20). Values are expressed as mean ± s.e.m. of three independent experiments. The data were analyzed using the one-way ANOVA test ($F_{3, 4}$ = 8.73, P < 0.001), *Post hoc* pairwise comparison were done using Bonferroni test. Labels with different letters are significantly different at P = 0.05. (**c**) Effects of asc-C9 at the same concentrations as produced by a natural chamber on the chemotaxis index of dispersal forth-stage nematode L_{IV} (n = 10).

Values are expressed as mean ± s.e.m. of three independent experiments. (**d**) Effects of asc-C9 at the same concentrations as produced by one newly eclosed adult beetle on the chemotaxis index of dispersal third-stage nematode L_{III} larvae (n = 10). Values are expressed as mean ± s.e.m. of three independent experiments. The significance level was set at α = 0.05 and were not significant.

Supplementary Tables

Supplementary Table 1. Abiotic conditions and beetle development at field sites.

	Annual average temp. (°C)	Annual average rainfall (mm)	Latitude, Longitude	Elevation	Number of PWN L_{III} larvae around each chamber	Percent pupation on 15-Apr. (%)	Emergence time of adults
Infested site 1 (Tianwang Mountain) Host: *Pinus tabuliformis* Carrière	13.9	500	N 33° 68′, E 109° 1′	1000 m	6879.3* ±34.09	19.02 ±0.86	30-Apr., 2013 30-Apr., 2014 1-May, 2015
Infested site 2 (Xiuping Mountain) Host: *Pinus tabuliformis* Carrière	13.4	741.9	N 33°25′, E 109° 8′	1300 m	8168 ±29.92	23.93 ±0.57	30-Apr., 2013 28-Apr., 2014 30-Apr., 2015
Uninfested site 1 (Yuan Mountain) Host: *Pinus tabuliformis* Carrière	12.2	804.4	N 33° 23′, E 109° 26′	850m	0	0	10-May, 2013 7-May, 2014 8-May, 2015
Uninfested site 2 (Fengtuling Mountain) Host: *Pinus tabuliformis* Carrière	12.8	804.8	N 32° 03′, E 109° 15′	830 m	0	0	7-May, 2013 9-May, 2014 10-May, 2015

* n=30 samples per group and three independent experiments.

Supplementary Table 2. Primers used for quantification of the specific mRNAs.

mRNA	Forward primers (5’-3’)	Reverse primers (5’-3’)
β actin	TGGGTATGGAATCTTGCGGT	GGCGGTGATTTCCTTTTGCA
Kruppel homolog 1 (Kr-h1)	TTGCCGCAAATCTCACAGGA	TTCGAACACTCCGGAAAGCT
Methoprene-tolerant (Met)	ACTGGGCGGTCGTTTTGTTA	ACCACCCAGAAGCAACGATT
HMG CoA synthase	TCACTCTCGTCACTTGCAGT	TTTCCCAGCTGACACTCCAT
EcR	TACGGACAACAGTTTCGCGA	ACACTTCTGCCGGACAATGT
USP	AGGCGCATAAACGGTGAGTA	ATTGGTCGCCGAACACATTG
E75	TCAGCGGTGTCAATGAACGA	TTTCGAGGCGGTTTGAATGC
insulin receptor 1	CAAGGCAGCTTTGGAATGGT	GGCAGTGTCAAAAGCCTTCA
insulin receptor 2	CCAATCTCCCATCAAAGGCG	TCGTCTGGATTTGAGCAGGT
c-Jun N-terminal kinase (JNK)	CAGTCTGATTCTGCGGTTGG	CCTACGTTCATCCGCAAGTG
vitellogenin 1	TGCTCAGAGGCTTCAGTGAA	CGCTTCCGTCAATTATCCGG
vitellogenin 2	AGGCCCTCTCTTTGAATCCC	TTCTGCATCCTCCCACCATT
carboxypeptidase	TGCCCGAACTCCCATACATT	AAGTTAGTCGCCGCCAAATC
CP AP1-2	TGCCGGTTCTGTTCTGTTTC	ACCATAGTCCCTGCCTTCAC
CP RR1-5	TAAGGCGAGGCTGTTTACCT	TGGTTCAGGAGCGTATGGTT
CP RR2-3	CCTCAAATCTCCTCCAGCCT	TGTTAAAGTGCGCGCTACTC
CP RR2-8	GCTGACGAGTTGATCACCAC	AACCACCAGCACCAAGAAAC
CP RR2-11	TCTTTGGCGGCTTTGACTTC	AGGCTGGAGGAGATTTGAGG
CP CFC-5	TGACCTACTCCATGGGCTTG	CAACAACGATGCCCAACAGA
Tweedle	TGATTCCACCGATTCCACCA	TCCCCATCGGCTATTCCTTC
